# The microbiome in urogenital schistosomiasis and induced bladder pathologies

**DOI:** 10.1371/journal.pntd.0005826

**Published:** 2017-08-09

**Authors:** Adewale S. Adebayo, Mangesh Survayanshi, Shrikanth Bhute, Atinuke M. Agunloye, Raphael D. Isokpehi, Chiaka I. Anumudu, Yogesh S. Shouche

**Affiliations:** 1 Cell Biology & Genetics Unit, Department of Zoology, University of Ibadan, Ibadan, Nigeria; 2 National Centre for Cell Science, Pune, India; 3 Department of Radiology, University of Ibadan, Nigeria; 4 Bethune-Cookman University, Daytona Beach, Florida, United States of America; George Washington University, UNITED STATES

## Abstract

**Background:**

Human schistosomiasis is a highly prevalent neglected tropical disease (NTD) caused by *Schistosoma species*. Research on the molecular mechanisms influencing the outcomes of bladder infection by *Schistosoma haematobium* is urgently needed to develop new diagnostics, therapeutics and infection prevention strategies. The objective of the research study was to determine the microbiome features and changes in urine during urogenital schistosomiasis and induced bladder pathologies.

**Methodology:**

Seventy participants from Eggua, southwestern Nigeria provided morning urine samples and were screened for urogenital schistosomiasis infection and bladder pathologies in a cross-sectional study. Highthroughput NGS sequencing was carried out, targeting the 16S V3 region. Filtered reads were processed and analyzed in a bioinformatics pipeline.

**Principal findings:**

The study participants (36 males and 34 females, between ages 15 and 65) were categorized into four groups according to status of schistosomiasis infection and bladder pathology. Data analytics of the next-generation sequencing reads revealed that Proteobacteria and Firmicutes dominated and had influence on microbiome structure of both non-infected persons and persons with urogenital schistosomiasis. Furthermore, gender and age influenced taxa abundance independent of infection or bladder pathology. Several taxa distinguished urogenital schistosomiasis induced bladder pathologies from urogenital schistosomiasis infection alone and from healthy persons, including known immune-stimulatory taxa such as *Fusobacterium*, *Sphingobacterium* and *Enterococcus*. Some of these significant taxa, especially *Sphingobacterium* were projected as markers of infection, while several genera including potentially beneficial taxa such as *Trabulsiella* and *Weissella*, were markers of the non-infected. Finally, expected changes in protein functional categories were observed to relate to cellular maintenance and lipid metabolism.

**Conclusion:**

The urinary microbiome is a factor to be considered in developing biomarkers, diagnostic tools, and new treatment for urogenital schistosomiasis and induced bladder pathologies.

## Author summary

The human microbiome comprises bacteria (plus viruses, fungi and archeae) inhabiting different sites of the body. They do not specifically cause diseases, but their presence, absence or population influence body functions. We therefore examined such organisms found along the urinary tract, in persons living in a rural community in Nigeria who considered themselves healthy, were infected with the parasite *Schistosoma haematobium* or had developed bladder complications along with the parasite infection. We found that these groups shared a large portion of the microbiome, but there were microbial species unique to infected persons and those with bladder complication. Some of these were capable of inducing inflammation and could offer less protection to the host. We also predicted pathways that are affected by the difference in the microbiome.

## Introduction

Human schistosomiasis is a devastating and highly prevalent neglected tropical disease (NTD) caused by Schistosoma species, a genus of parasitic flatworms with life cycle forms found in freshwater, freshwater snails and human organ systems [1]. Cercarial forms of the parasite are released from infected water snails and penetrate human skin. Eventually, the adult forms inhabit and produce eggs in the intestinal or bladder tissues. The cercariae, schistosomulum and adult stages are all capable of inducing host immune response [1–3]. The eggs of *Schistosoma haematobium* in the human bladder can cause a spectrum of urogenital clinical presentations including granulomatous inflammation, fibrosis, urinary tract infections and bladder cancer [2,3]. Seventy-eight countries and a quarter of a billion persons are at risk of schistosomiasis [4]. After considering the disease along with other NTDs, Nigeria was described as ‘ground zero’ of schistosomiasis due to the high endemicity in the country [5]. Few studies have reported on the immune response to urogenital schistosomiasis in Nigeria [6], with several studies in different parts of the country reporting prevalence rates between 15–57%[7, 8]. The occurrence and different forms of bladder tumors and bladder pathologies have been associated with urogenital schistosomiasis in Nigeria[9]and in other parts of Africa[2,10].

Research on molecular pathology influencing the outcomes of bladder infection by *Schistosoma haematobium* is urgently needed to develop new diagnostics, therapeutics and infection prevention strategies[3].Earlier studies have suggested that the mechanism of formation of bladder tumours will be due to formation of nitrosamines, polyaromatic hydrocarbons, free radicals, and presence of microbes [11]. More recently, studies have highlighted the role of estrogen-related molecules from the parasite in disease progression [12], based on the discovery that they could be oxidized to form adducts [13], induce infertility in females [14], and could probably induce error-prone DNA repair [15]. Molecules related to estrogen were recently detected in urine samples of persons infected with schistosomiasis [16].

Our collaboration with communities in Nigeria at risk of schistosomiasis bladder-related pathologies presents opportunities to conduct basic and translational research projects[6,9,17]. We are interested in understanding the influences of microbial taxa on human health and disease [18] including the induction of bladder pathologies in urogenital schistosomiasis. Genomic sequencing technologies can determine the membership of, and functions performed by microbial communities (microbiome) in the urine [19–21]. The human microbiome is the community of microbes that is estimated to encode up to several million genes with the capacity to influence human health and disease[22]. The microbiome mediates the effectiveness of drugs, xenobiotics and vaccines[23], and influences disease or health status [24].

The microbiome along the urinary tract structure can have adverse or beneficial effects on human health [19–21]. Therefore, the goal of the research study reported in this article was to understand the microbiome features (taxa membership and functions of encoded microbial proteins) in the urine samples from healthy persons and persons infected with urogenital schistosomiasis and related pathologies.

The availability of volunteers with asymptomatic schistosomiasis in Eggua, in southwestern Nigeria allowed us to accomplish the research goal. Seventy volunteers were categorized into groups based on the presence of infection and induced bladder pathologies. The objectives of the study were to (1) determine the microbiome changes in urine samples during urogenital schistosomiasis and induced pathologies; and (2) identify functional biological processes that could be altered by such changes.

## Methods

### Overview of methods

The approach of the research study consisted of recruiting study participants; screening for schistosomiasis infection and bladder pathologies; microbiome sequencing; pre-processing of microbiome sequences; and data analytics of microbiome sequence data collection.

### Ethics and study participants

Study participants were adults recruited from Eggua, in Ogun State, southwestern Nigeria (07° 01.592 N; 002° 55.083 E) in a cross-sectional study. Nearby communities have previous history of urogenital schistosomiasis and it was shown recently that the bladder pathologies occurrence in these localities is essentially driven by prevalence of urogenital schistosomiasis infection [7]. Ethical approval was obtained from University of Ibadan/University College Hospital Institutional Review Committee as well as the Ogun State Ministry of Health. Participants were duly informed about the study before sampling and informed consent obtained from all participants, with language translation as required. Exclusion criteria included recent use of antibiotics, painful bladder, urine discharge problems, common urinary tract infection. This was determined by interviews. While some of these criteria may indeed be due to urogenital schistosomiasis, it was necessary to prevent confounders and ambiguity. Samples suspected of urinary tract infection (positive nitrites, positive leukocyte esterase and ≥ 5wbc/high power field) were removed from further analysis. Seventy samples meeting the exclusion criteria were eventually processed for high throughput sequencing.

### Screening for infection and bladder pathologies

Medical history, routine diet and demographic factors were obtained via structured questionnaire, and participants provided midstream urine samples. All samples were collected in the morning hours and immediately anonymized upon collection. The presence of analytes in the urine samples was immediately determined with Urinalysis Reagent Strips (Rapid Labs, UK), and urine microscopy was done on a 10 ml aliquot to detect *S*. *haematobium* eggs after sedimentation; and the rest was immediately frozen until further use and transported under dry ice conditions when required. Egg shedding may be infrequent in chronic infection, hence if a sample was negative for *S*. *haematobium* eggs, it was subjected to PCR detection using published *Dra1* primers [25]. Briefly, 25ul PCR reaction mix containing 100ng isolated urine DNA was prepared. Cycling conditions were: initial denaturation at 95°C for 5mins, 30 cycles of 95°C for 30 secs, 55°C for 30 secs and 72°C for 1min, and final extension at 72°C for 10mins. Bladder scans were carried out with Titan UltraSystem (Sonosite, WA, USA) by a trained radiologist, and all images scored according to WHO recommendations [26] and also anonymized.

### Sequencing and pre-processing

One ml of urine was pelleted and DNA was isolated using Qiagen Blood and Tissue kit (Qiagen, Hilden, Germany), with the modification of adding 20mg/ml of lysozyme at the lysis stage. Isolated DNA was quantified with NanoDrop spectrophotometer (Thermo Fisher Scientific, MA, USA), with quality assessed as 260/280 absorbance ratio >1.8. Library preparation and sequencing were done as previously described [24]. Briefly, quality control of sequencing library (size and quantity) was done on 2100 BioAnalyzer (Agilent Technologies, CA, USA) following manufacturer’s protocol. Highthroughput sequencing targeting the V3 region of the 16S rRNA gene was carried out on IonTorrent PGM platform. Sequencing and barcoding were done using Ion PGM Sequencing 200 kit v2 and Ion Xpress Barcodes Adapters (Thermo Fisher Scientific, MA, USA) using manufacturer's protocol. Barcoded libraries purified with Agencourt AMPURE beads (Beckman Coulter, CA, USA), and equimolar amplicons were pooled prior to sequencing. V3 primers used were 343F- 5’TACGGRAGGCAGCAG3’and 533R- 5’TTACCGCGGCTGCTGGCAC 3’. Processing and quality filtering of sequence data was done using QIIME 1.9.1 [27]. Operational Taxonomic Units (OTUs) were defined based on 97% sequence similarity and taxonomic classifications were assigned using the Greengenes g_13_8 [28]. Where reference to the Greengenes database did not identify OTUs up to genus level, we used BLASTn to assign taxonomy based on 100% coverage and >98% identity, though this was possible for only two OTUs. Sequence data was examined for possible contamination from kits using the correlation between OTUs and amplicon concentration (purified individual library) [29, 30]. Sequencing was evaluated with Good’s coverage and rarefaction plots.

### Data analytics of microbiome sequence collection

Data was screened for missing values, normality and excessive outliers before applying statistical tools. After rarefaction to 2952 reads, biostatistical analyses were performed using STAMP [31], R packages Vegan [32] and Biom [33]. Public server at http://huttenhower.sph.harvard.edu/galaxy/ was used for LEfSe [34]. All tests were two-tailed; significant results were indicated by a p value < 0.05 and effect size estimated with Eta-squared. Mann-Whitney test (or non-parametric White's test [35] in STAMP) was used to test for significant differences between two groups. Comparison of multiple groups was done using non-parametric Kruskal-Wallis test and Tukey-Kramer post hoc test. Benjamini-Hochberg test/FDR was used for multitest correction. In consideration of the criticisms of normalization using rarefaction [36], which in this case would leave out 11 samples from analysis, we applied edgeR’s RLE method [37] to normalize samples, for the fold change or differential abundance analysis of microbiome; thus including all 70 samples. Variance threshold was set at 1e-5 and FDR<0.05. Functional profiles were inferred with PICRUst [38].

## Results

### Overview of results

One of the key findings of this research was that microbial taxa membership and predicted protein function were uniquely discriminant for persons with urogenital schistosomiasis and those who were not infected. Data and project information are deposited in NCBI’s Sequence Read Archives under accession SRP094688. A total of 1.4 million non-polyclonal, trimmed and filtered reads was obtained after quality control. Good’s coverage was 98% and average conditional uncovered probability estimates was 0.05. The reads formed 2946 OTUs, excluding 1504 singletons which were removed before abundance and multivariate analyses. No OTUs were strongly and negatively correlated with purified amplicon concentration, but ten OTUs had moderate, negative correlation (rho = -0.3 to -0.4). This indicated that there was minimal level of probable contaminants from reagents. About 110 OTUs occurred in at least 55% of samples and only 1 OTU in all samples.

### Seventy volunteers categorized into four groups according to status of infection and bladder pathology

The seventy samples analysed for urinary microbiome through NGS comprised 36 males and 34 females, between the ages of 15 and 65 years. Interviews revealed that the diet was essentially uniform, with a strong starch base. Bladder pathologies detected included abnormal thickness (>5mm of a full bladder), calcification, bladder mass, hyperplasia and irregular shape. We identified four major groups of participants based on the status of infection and bladder pathology (Table 1), comprising (a) the Advanced group, having infection and bladder pathologies, (b) Bladder pathology-only group, (c) infection-only (detected by microscopy or PCR), and (d) controls (no infection or pathology). For microbiome abundance analysis, we also grouped all samples into two—infected and non-infected.

**Table 1:** Participants in urine microbiome study from Eggua, southwestern Nigeria.

GROUPING	CATEGORIES	SAMPLE COUNT (PERCENTAGE)
**AGE**	ELDERLY (50years +)	25(36)
	MID-AGED (30–49 years)	30(43)
	YOUNG (<30years)	15(21)
**INFECTION**	NON-INFECTED	24(34)
	INFECTED	46(66)
**DISEASE PROGRESSION**	ADVANCED	22(31)
	PATHOLOGY-ONLY	10(14)
	INFECTION-ONLY	25(36)
	CONTROL	13(19)

Advanced, urogenital schistosomiasis infection + abnormal bladder morphologies

Infection-only, urogenital schistosomiasis infection

Pathology-only, abnormal bladder morphologies

Controls, no infection or pathology.

### Proteobacteria and Firmicutes dominate and influence urine microbiome structure

**Fig 1:**
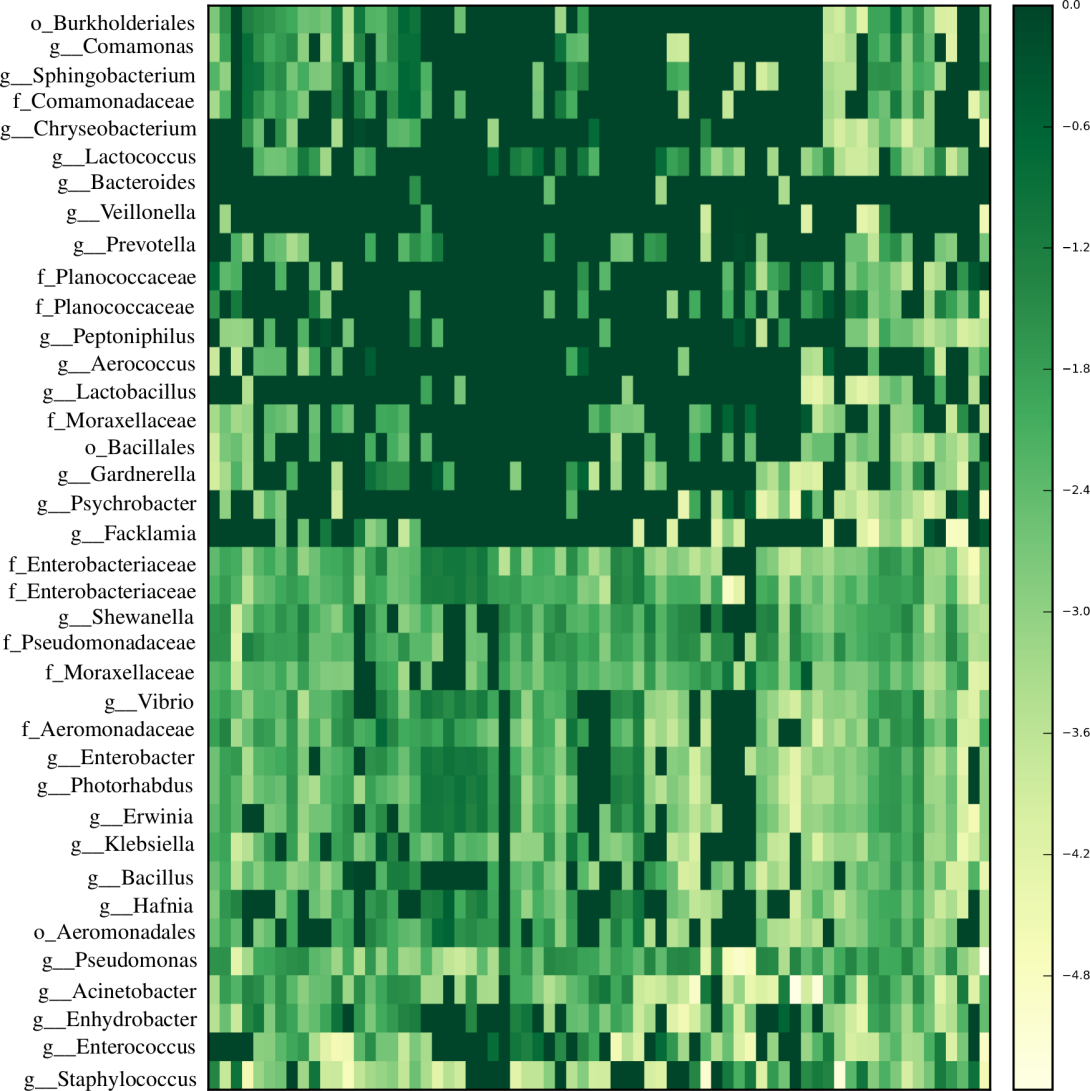
The most dominant genera and their respective families in the urinary tract microbiome of individuals in Eggua, southwest Nigeria. Dark colors represent lower abundance, lighter colors represent higher abundance.

**Fig 2:**
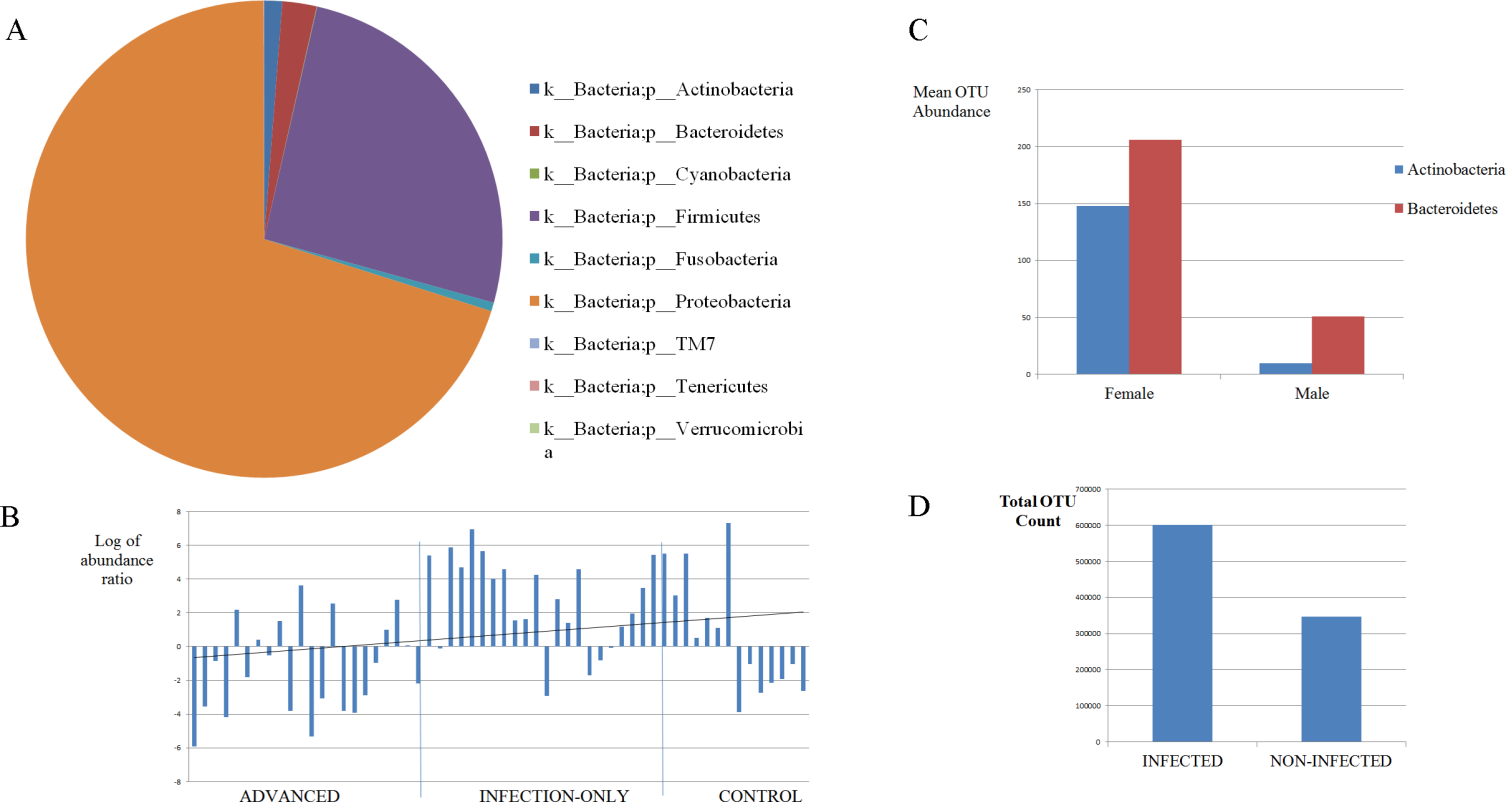
(A) Mean relative abundance of various phyla in the urine microbiome of a study population in Eggua, southwestern Nigeria. (B) Bar plot of the Firmicutes/Proteobacteria log abundance ratio in different states of urogenital schistosomiasis. The correlation was not significant (p = 0.08). (C)Abundance of phyla Actinobacteria and Bacteroidetes with regard to gender in the urine microbiome of a study population in Eggua, southwestern Nigeria (p<0.05). (D) Proportions of filtered OTU counts in urogenital schistosomiasis and controls(p = 2.06E-16).

Differences in proportions of some phyla in the urine microbiome in the study population are presented in Figs 1 and 2A–2D. The mean relative abundance of two phyla, Proteobacteria (70%) and Firmicutes (26%) were the largest in the microbiome of the sample population, with Bacteroidetes, Actinobacteria, Fusobacteria, Cyanobacteria and others making up the rest (Fig 1, Fig 2A). The proportions of Proteobacteria differed among groups, highest in infection-only (65%) and pathology-only (80%) cases, and lowest in advanced cases (42%). The Proteobacteria proportion was higher in non-infected (73%) compared to infected (66%), and was unchanged in pathology (68%) compared to pathology-absent cases (69%). In all three comparisons, the opposite is true for Firmicutes. Virtually all of the Actinobacteria OTUs were found in controls and advanced cases. Having observed increased Firmicutes and decreased Proteobacteria in infected cases, the log of the ratio of the abundance of Firmicutes to Proteobacteria was calculated for each sample and plotted as a dysbiosis index. This index associated negatively with disease progression but the correlation was not statistically significant (rho = -0.29, p = 0.08) (Fig 2B). The most dominant genera were *Pseudomonas*, *Staphylococcus*, *Acinetobacter*, *Enterococcus*, unclassified Enterobacteriaceae and *Facklamia* (Fig 1). The first three, made up 60% of the whole community. Of the OTUs formed, the most abundant OTUs were those belonging to *Pseudomonas* and *Staphylococcus* (Fig 1). *Facklamia* was essentially found only in advanced cases, making up 12% in proportion. *Pseudomonas* proportions were increased by more than two-fold in infection-only and pathology-only cases (each at 47%) compared to advanced cases (19%) or controls (15%).

**Fig 3:**
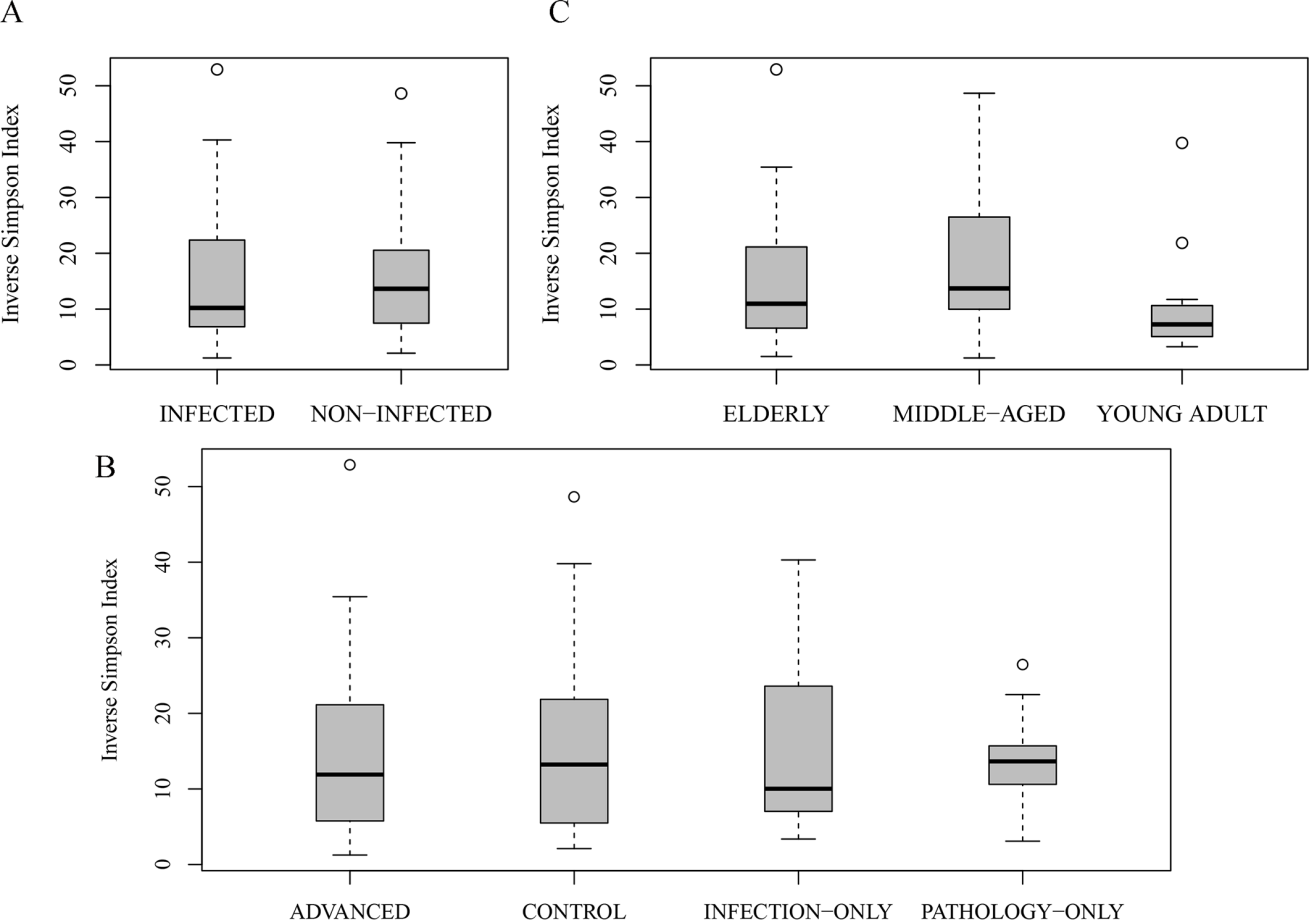
Comparison of microbiome diversity indices (A) between urogenital schistosomiasis infection and controls, (B) in different states of urogenital schistosomiasis and controls, and (C) among age groups. Dark band represent mean diversity index, circles represent outliers. The differences in diversity were not significant (p>0.05) except in C(p = 0.038).

In terms of diversity of the samples, alpha diversity was estimated using the inverse Simpson’s index (1/D); this measure takes both OTU richness and relative abundance into account and the results are presented in Fig 3A–3C. We found a non-significant reduction in mean diversity in infected cases compared to non-infected, in pathology cases compared to no-pathology cases, and in advanced cases compared to others (p = 0.1) (Fig 3A and 3B). There was higher number of OTUs detected in infected, pathology and advanced cases, respectively in each case. This indicated that advanced cases had higher number of OTUs but reduced diversity index compared to the other three groups. The same was observed for infected cases compared to non-infected cases.

**Fig 4:**
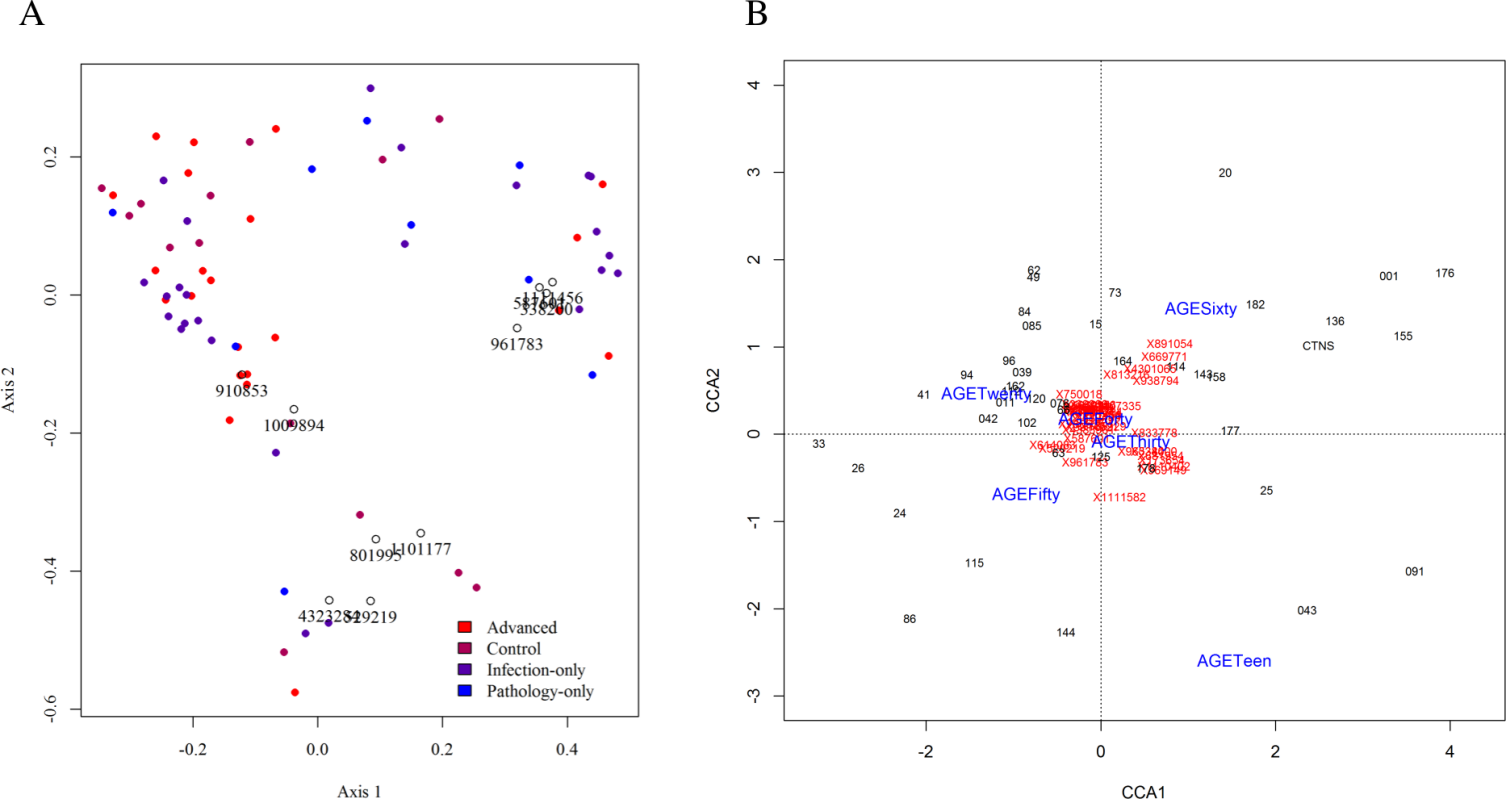
(A) Principal Coordinates biplot of beta diversity in different states of urogenital schistosomiasis and controls. Circle and small typeset represent Greengenes ID of dominant OTUs. These dominant OTUs were mainly assigned to *Pseudomonas* and *Staphylococcus*, but had little influence on disease status. (B) Partial canonical correspondence analysis of age groups without the influence of urogenital schistosomiasis infection. Blue fonts are age categories, red typeface are Greengenes ID of the most influential OTU. Clustering together indicates no special correlation with age group. OTUs that corresponded closely with participants aged sixty years were mainly assigned to *Acinetobacter*.

Beta diversity was assessed using Bray-Curtis metrics to estimate dissimilarities based on OTU level, rarefied data. Similar patterns were observed considering infection and pathology. The first two principal coordinates explained 36% of the variation among them, and a plot of the first two axes revealed little level of axes separation among the sample groups (Fig 4A). Of the ten most dominant OTUs, 4 of them, all assigned to the genus *Pseudomonas*, were prevalent (and in opposition to other prevalent OTUs) along the first axis (Fig 4A). They clustered close to several infected or pathology samples. Four other OTUs, all assigned to *Staphylococcus* were in contrast, driving the axes in the opposite direction, and clustering close to some control samples (Fig 4A).

### Gender and age could influence taxa abundance independent of infection or bladder pathology

Mean diversity of organisms differed among age groups (Fig 3C), and the middle-aged group contained 33% more OTUs than the young or elderly age groups. Since presence of infection or pathology could be expected to affect taxa differences in different ways among age groups and prevalence rates were different among the age groups, we tested taxa differences among age groups using only controls (n = 13), There were no significant taxon differences at all levels tested, which was probably due to low sample size for comparison. We therefore used partial canonical correspondence analysis to explain taxa differences with age of participants. This will involve all samples and statistically exclude the influence of being infected or having pathology. To achieve this, the age groups were arranged into smaller ranges i.e. age 10–20 (teens), 20–30, up to 60 and above. This was to increase the number of age groups to six, which was considered better in this type of analysis than having three groups. Canonical correspondence analysis was then mapped using the age ranges as constraining variables and infection as conditioning variable. This was done in order to parse the effect of being infected or not on each taxon. Only OTUs present in at least 40% of samples were utilized. Four OTUs, all assigned to genus *Acinetobacter* appear sensitive to age 60 and above, those assigned to *Citrobacter*, *Enterococcus*, and Enterobacteriaceae were sensitive to age 30–40 (Fig 4B). Indeed, age group 30–40 could be influenced by many OTUs. Similar results were obtained with pathology as the conditioning variable.

There was a difference between sexes in the microbial community at the OTU and genus levels. There was more abundance of Actinobacteria and Bacteroidetes phyla in females than males (Fig 2C). More heterogeneity was observed in females, with 40% more OTUs present compared to males. There was a non-significant reduction in diversity in female samples. Using only control samples, unclassified Enterobacteriaceae and unclassified Pseudomonadaceae were significantly abundant in females (p = 0.035, p = 0.021, respectively).

### Several taxa distinguish urogenital schistosomiasis infected cases from non-infected cases, as well as advanced cases from infection alone

**Fig 5:**
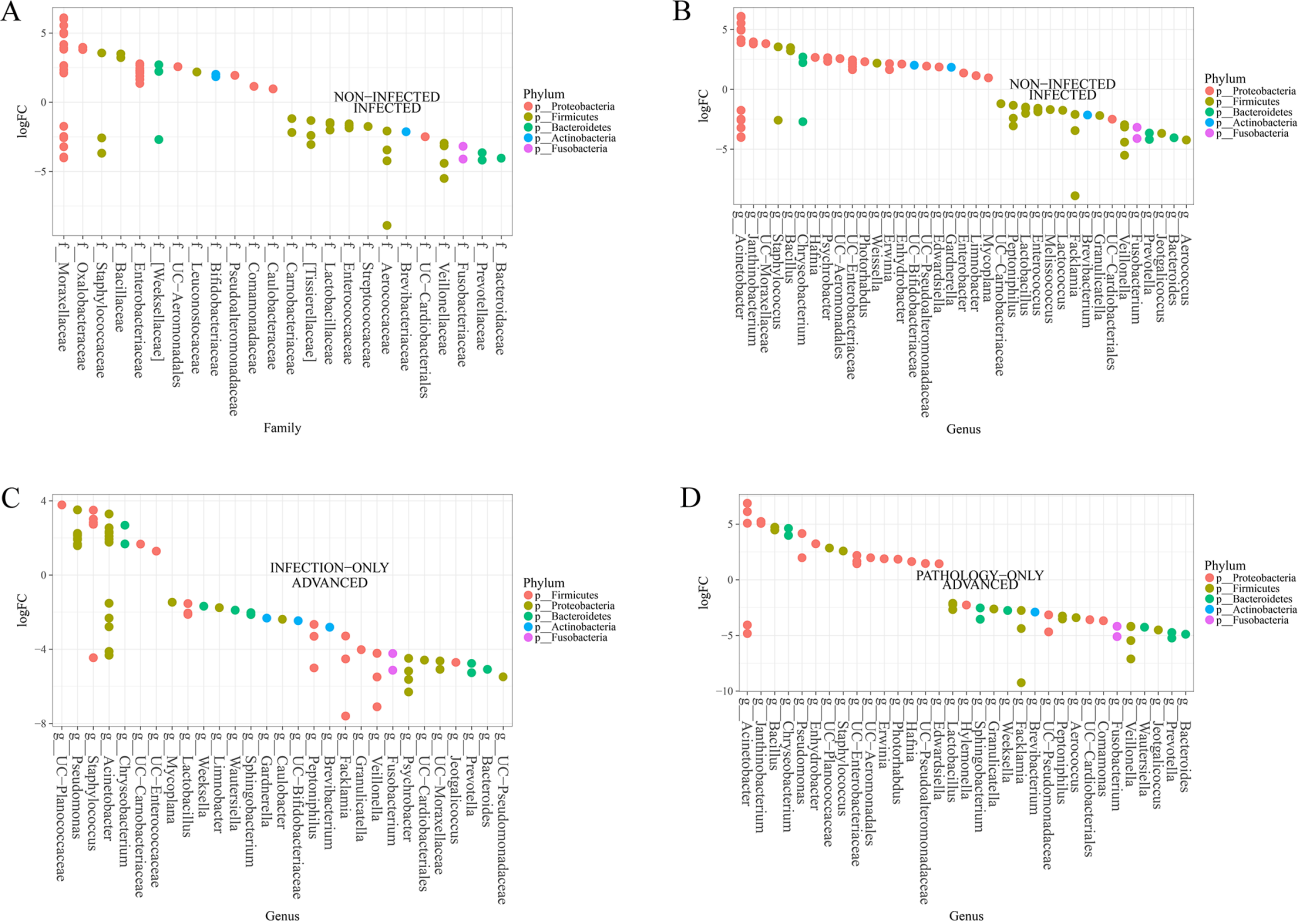
Specific urinary microbes are abundant in different states of urogenital schistosomiasis infection. (A) Differentially abundant microbiome families in urogenital schistosomiasis (infected) and controls (non-infected), (B) Differentially abundant microbiome genera in urogenital schistosomiasis (infected) and controls (non-infected), (C) Differential microbiome genera between urogenital schistosomiasis (infection-only) and urogenital schistosomiasis induced bladder pathology (advanced), (D) Differentially abundant microbiome genera in non-infected bladder pathology (pathology-only) and urogenital schistosomiasis induced bladder pathology (advanced) (FDR<0.05). Differential abundance was measured with LogFC, the log2 of the number of times the OTUs belonging to a genus (or family) are more numerous in one group relative to the other. Circle on a vertical line represents a bacterial genus or family colored by their phylum and the genus or family is named at the end of the line. More than one circle on a vertical line represents species of the same genus. The genus is labeled on the x-axis. UC represents a genus whose identity could not be completely confirmed, but with known family or order. Abundant microbes in each of the two groups are presented on either side of the middle zero line.

There was a significant difference in the microbial community between infected and non-infected, at all levels tested, genus level (p = 6.55E-10) and OTU levels (p = 2.06E-16) (Fig 2D). Several features (taxa) could distinguish persons infected with urogenital schistosomiasis and non-infected persons. Differences in the abundance of microbiome taxa in the study groups are depicted in Fig 5A–5D, S1 Fig and S2 Fig. At phylum level, the differences in the dominant phyla, Proteobacteria and Firmicutes, was not statistically significant between the infected and the non-infected. Phylum Fusobacteria, was the only significant phylum (FDR = 0.006), to distinguish infected samples from non-infected samples. At family level, twenty-two families showed differential abundance between infected and non-infected. The abundance of *Veillonella*ceae, Fusobacteriaceae, Lactobacillaceae and Enterococcaceae were among those significantly higher in infected cases, while Oxalobacteraceae, Enterobacteriaceae, Staphylococcaceae among others were significantly abundant for non-infected samples (FDR<0.05) (Fig 5A). At the genus level, bacterial genera most significantly abundant in infected samples included *Facklamia*, *Fusobacterium*, *Veillonella*, *Bacteroides*, *Lactobacillus* and *Enterococcus*. On other hand, *Bacillus*, *Staphylococcus*, *Janthinobacterium*, *Edwardsiella*, unclassified Bacillaceae, unclassified Enterobacteraceae, *Trabulsiella*, *Xenorhabdus*, *Collimonas* and *Weissella* were significantly associated with non-infected samples (FDR<0.05) (Fig 5B). Different *Acinetobacter* OTUs were associated with both infected and non-infected samples (Fig 5B). Only some of these genera, especially *Fusobacterium* and *Janthinobacterium*, were found to be highly abundant in infected or non-infected samples, respectively, if rarefaction was applied before analysis (S2A Fig).

Comparing controls to other groups, unclassified Clostridiales (p = 0.047, η^2^ = 0.11) was highest in controls, and lowest in infection-only and pathology-only cases (S1A Fig). Enterobacteriaceae (0.24≥ η^2^ ≤0.29, 0.022≥ p ≤0.039) was more abundant in controls (S1B Fig), and Pseudomonadaceae (0.14≥ η^2^ ≤0.31, 0.025≥ p ≤0.048) far more abundant in infection-only cases and pathology-only cases (S1C Fig). The correlation of *Lactobacillus* abundance and healthy status was negative (rho = -0.34, p = 0.039) (S1D Fig).

**Fig 6:**
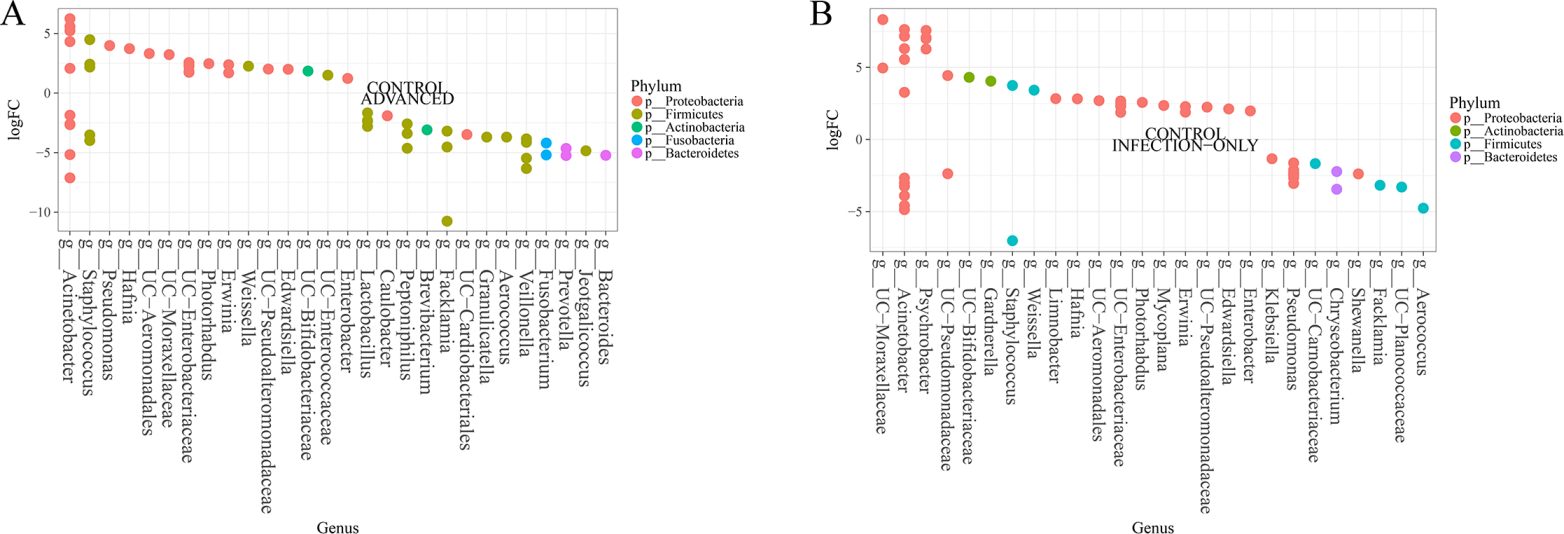
Urogenital schistosomiasis and induced bladder pathology result in specific microbiome changes in the urinary tract. Differentially abundant microbiome (A) between urogenital schistosomiasis induced bladder pathology (advanced) and healthy controls, and (B) between urogenital schistosomiasis (infection-only) and healthy controls (FDR<0.05). Differential abundance was measured with LogFC, the log2 of the number of times the OTUs belonging to a genus (or family) are more numerous in one group relative to the other. Circle on a vertical line represents a bacterial genus or family colored by their phylum and the genus or family is named at the end of the line. More than one circle on a vertical line represents species of the same genus. The genus is labeled on the x-axis. UC represents a genus whose identity could not be completely confirmed, but with known family or order. Abundant microbes in each of the two groups are presented on either side of the middle zero line.

Five genera—*Facklamia*, *Veillonella*, *Fusobacterium*, *Bacteroides*, and *Aerococcus*—were the most differentially abundant in advanced cases, when compared with infection-only cases, pathology-only or control cases (Fig 5C and 5D, Fig 6A). *Aerococcus*, *Acinetobacter* and *Staphylococcus* were most changed in infection-only cases (Fig 6B, S2B Fig), though other OTUs of *Acinetobacter* and *Staphylococcus* were highly associated with control cases. The least changes in taxa between groups were in the pathology-only: control pair and the pathology-only: infection-only pair.

### Linear discriminant analysis prediction highlighted fewer taxa as biomarker

**Fig 7:**
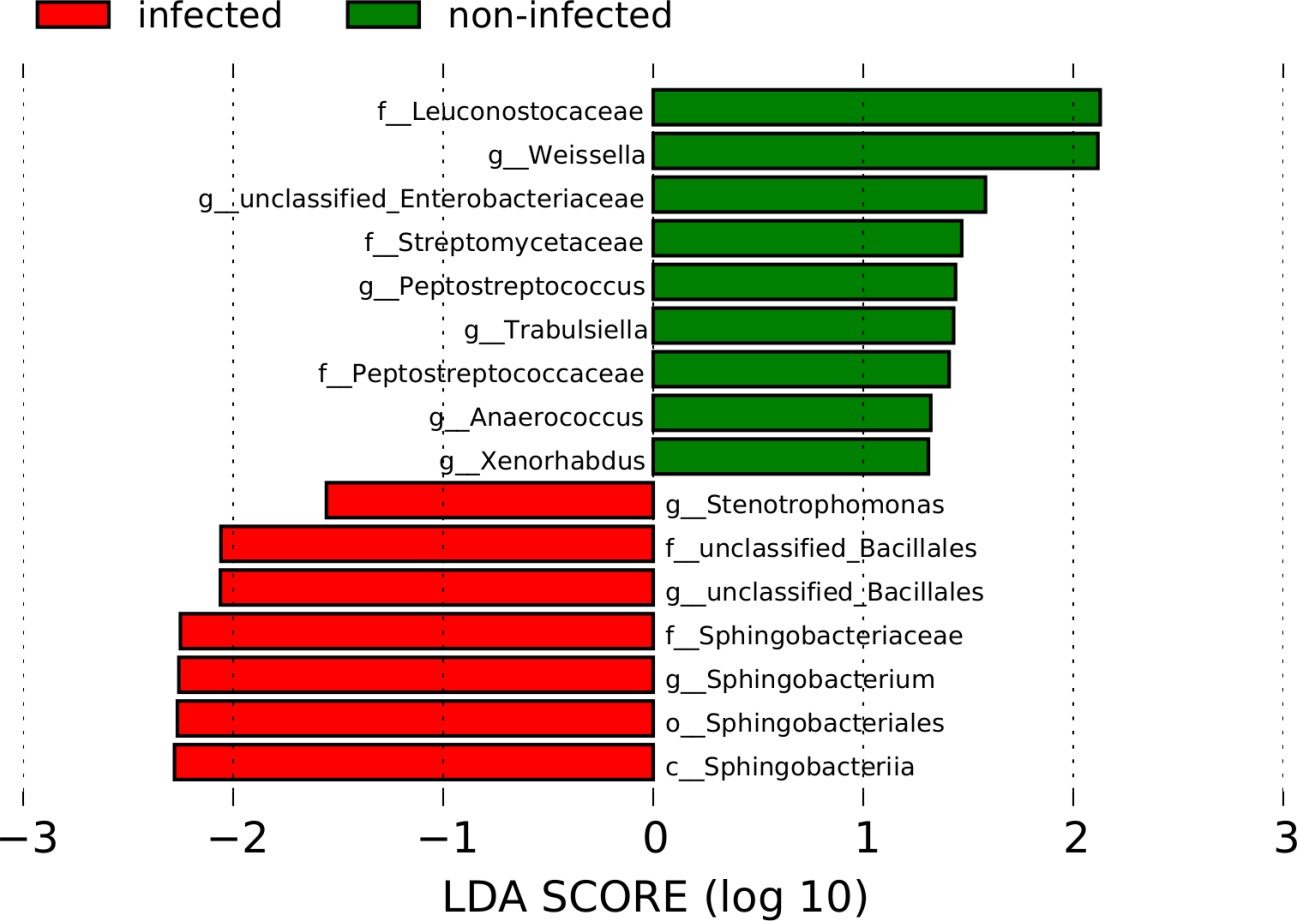
Predicted microbial biomarkers in urogenital schistosomiasis cases (infected) and controls (non-infected).

Microbiome sequence data was subjected (at OTU and genus levels) to discriminant analysis using LEfSe to identify possible biomarkers. Comparing infected and non-infected cases, all taxa that were significantly associated with each group from earlier significance analysis were also identified at the OTU level. At genus levels, only two of the significant taxa, *Sphingobacterium* and *Aerococcus* were projected as markers of infection, while several genera including *Trabulsiella* and *Weissella*, associated with non-infected (Fig 7).

### Predicted changes in functional categories relate to cellular maintenance and lipid metabolism

Using our sequence abundance data, we obtained imputed metagenomes and the associated KEGG Orthology pathways present in the microbiome. Nearest Sequenced Taxon Index (NSTI) summary ranged from 0.021 to 0.053 (mean = 0.039). This indicated that several closely related genomes were available for inference purposes. A total of 328 functional categories from 6908 KOs were identified.

**Fig 8:**
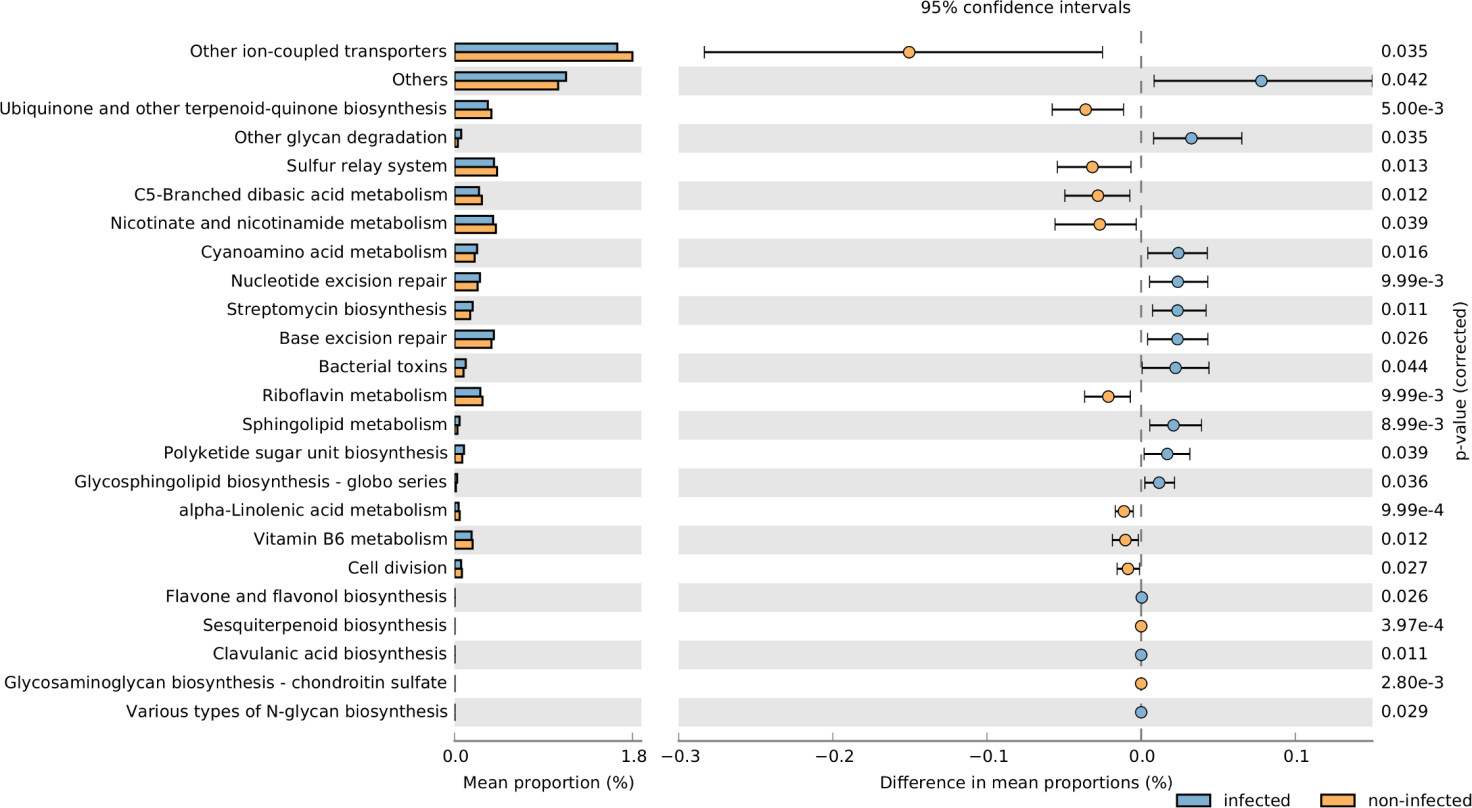
Predicted biochemical changes in the KEGG Ortholog database due to microbiome differences between urogenital schistosomiasis cases (infected) and controls (non-infected).

Of the features differentiating infected samples, several were significant (Fig 8) between infected and non-infected cases, though the effect sizes were rather small. This may be expected given that large portions of microbiome were shared. Considering all four groups together, 7 features which were statistically different—including lipid metabolism (reduced in advanced cases) and alpha-linoleic acid metabolism (reduced in advanced and infection-only cases) (0.1<η^2^ >0.143). In pairwise comparison with other groups, the more abundant features with the best effect sizes in advanced cases were pathways involving DNA maintenance including mismatch repair (η^2^ = 0.28), DNA recombination/repair, and translation factors (S3 Fig).

## Discussion

The overall microbiome structure from our data is, in part, similar to those of earlier studies [19, 20, 39, 40]. These studies also showed that the two phyla, Proteobacteria and Firmicutes, which form the core of the microbiome in the present study, occur in human urinary microbiome. But, in contrast to the aforementioned studies, low proportions of Firmicutes, very high proportion of Proteobacteria, and very low proportions of Actinobacteria /Bacteroidetes, are the features which clearly differentiate the microbiome data of the present study. The greater abundance of Actinobacteria and Bacteroidetes in females as reported previously [41], was also observed in the current study. Despite the low occurrence of these two phyla, the number of their OTUs in the sequence data of the present study was at least three times higher in females than males in this study (Fig 2C). In addition, the dominance of the Proteobacteria and Firmicutes was found in all samples (Fig 1, Fig 2A), unlike in the previous reports mentioned [19, 20, 39] where only few samples had such dominance. The study location is probably of importance in these contrasts; the present study involves a wholly rural and African population, while all these aforementioned reports have been European, Australian or North American. This raises the question of whether a large reduction in Firmicutes and consequent increase in Proteobacteria could be characteristic of a rural African urinary microbiome. Also, the higher proportion of Proteobacteria in non-infected cases, and its lower proportion in advanced cases compared to infection-only or pathology-only, indicates that the dominance of Proteobacteria in our population is not linked with disease conditions.

Another observation of the microbiome structure is the relatively high number of singleton OTU (1504 out of 4451). This may be indicative of considerable level of individual uniqueness in urinary microbiome composition, though it is also possible that a greater depth of sequencing will reduce the number of singletons. Such an indication of individual uniqueness is buttressed by the nature of the principal coordinates plot (Fig 4A). The spread of the coordinates indicates that there was substantial within-group variation, which therefore affected between-group axis separation. With this, our data shows there is individual uniqueness in the urinary tract microbiome composition.

In a study [42], two of the most dominant genera from our data, *Pseudomonas* and *Staphylococcus*, were isolated in only 2% and 18%, respectively, of 920 school children infected with urogenital schistosomiasis in Ibadan, southwestern Nigeria [42]. The study, which was entirely culture based, also highlighted that common urinary tract infection dominated by *E*.*coli* and *Klebsiella* may associate with urogenital schistosomiasis infection. In the current study, we systematically removed samples suspected of UTI, in order to focus on urogenital schistosomiasis alone and to reduce the possibility of analyzing co-infected samples. In addition, we used a high throughput DNA sequencing approach. Thus, our study was different and shows that *Pseudomonas* and *Staphylococcus* species are widespread in the population of interest and not simply because of infection.

There was a trend of reduction in the mean diversity of the microbiome in infection and advanced cases, and previous studies have reported such a reduction in diversity in disease conditions; such as in cystitis [43] and in inflammatory bowel disease [44]. In the current study, the reduction in diversity is most likely due to presence of *S haematobium* infection because there is increased diversity in pathology-only cases (n = 10). Hence the reduction in diversity in advanced cases is not likely to be because of the contribution of pathologies, but rather of *S haematobium* infection. The observation of a reduction in diversity, coupled with increased number of OTUs, may indicate that almost all species in the urine microbiome were covered by the sequence data and an increase in total sequence collection may not substantially increase microbiome diversity. For age groups and gender, higher number of OTUs was observed in middle aged and in females, indicating greater heterogeneity in females and the middle-aged. This heterogeneity is not due to infection or pathology because in terms of gender, males had more infection and pathology than females, and the middle-aged group had similar infection or pathology rates with other groups.

Associating specific taxon with disease state is an important goal in microbiome studies. For this purpose, the results of the present study were mainly examined at the family and genus levels. A substantial number of abundant genera are pathobionts, including–*Enterococcus*, *Bacteroides*, *Facklamia* (increased in infected) and *Enterobacter*, *Chryseobacterium*, *Edwardsiella*, etc (increased in controls). However, based on literature and our functional analysis, several of the dominant genera are biologically relevant within the environment. Some of them have been previously found in association with a non-healthy status. *Enterococcus* was associated with neuropathic bladder patients who are at risk of asymptomatic bacteriuria [45]. A previous study [20] identified *Pseudomonas* (in males only), *Staphylococcus* (in both genders), *Enterococcus* and *Facklamia* (females only) in urine samples of study subjects who were ‘partially healthy’ i.e. without any urinary tract disease or symptoms, but having other non-specified ailments. Also, their individual attributes as a result of specialty genes, proteins or pathways could be of importance in the urinary microbiome, as discussed subsequently.

Clearly, the dominant genus, *Pseudomonas*, in the microbial community did not significantly differ among groups and it may then be suspected that the genus had no influence on urogenital schistosomiasis or induced bladder pathologies, especially, since the *Pseudomonas* genus is a known opportunistic pathogen in common urinary tract infections [46]. However, a point of interest is the ability of some strains, including *P*. *fragi and P*. *putida* (along with very few Proteobacteria strains), to utilize extraneous steroids such as estrogen, due to the presence of specialized genes, in particular, tesD [47]. This is of interest because a recent report indicated the availability of catechol estrogens, steroid-like molecules, in the urinary tract during urogenital schistosomiasis infection [16]. In the present study, apart from being a dominant genus, the proportions of *Pseudomonas* and *P*. *fragi* were higher in infected cases. Some other OTUs assigned to the same genus were also significantly higher (0.00799> p<0.042). We hypothesize that the presence and proportion of some strains of *Pseudomonas* may be linked to the availability of greater amounts of steroid-related lipids or other lipid metabolites in the urine of infected persons and those with induced pathologies. Such a hypothesis requires further investigation involving gene expression studies, or reporter assays and strain identification for evaluation and confirmation.

Still on the dominant genera, the presence of different sets of OTUs of *Acinetobacter* or *Staphylococcus* in infected or advanced cases and in non-infected controls indicate a species-specific abundance in the two conditions. It is difficult to delve deeper into such observation, because longer length sequences than those used in this study are needed to confidently identify species-specific difference in the two opposing conditions. Nevertheless, it is known that some *Acinetobacter* species, for instance, *A*. *johnsonii* can destroy worms while others are pathobionts [48].

We found that genera with roles in the inflammatory process of human and other mammalian hosts associated strongly with and were differentially abundant in infected and advanced cases. The genus *Fusobacterium* (up to family, order and phylum level) consistently associated with advanced cases but not infection-only cases. *Fusobacterium* possesses potent lipopolysaccharides, are known to recruit immune cells [49] and are ubiquitous in colon cancer biopsies [50]. The genus is also known to serve as anchor for biofilm formation [49]. *Sphingobacterium*, identified as a marker in urogenital schistosomiasis, is known to possess unusual, non-mammalian sphingolipids containing ceramides. Such compounds are immunogenic and can activate macrophages, thereby contributing to the inflammatory process [51]. Human IgE antibody regulation could be affected in allergy or atopy, by *Enterococcus* [52,53]. We found elevated proportions of *Enterococcus* in infected persons, and increased IgE levels were associated with urogenital schistosomiasis [54]. It raises the question as to whether *Enterococcus* prevents excessive buildup of IgE in urogenital schistosomiasis infection. Thus, a future research objective would be to determine the influence of *Enterococcus* species on human IgE antibody production in urogenital schistosomiasis. To add to this, *Bacteroides* numbers are sometimes correlated with inflammation, and its lipopolysaccharides are potent immune-stimulators, yet its polysaccharide A is also capable of repressing pro-inflammatory cytokine [55, 56]. The four genera discussed above were differentially abundant in advanced cases, implying that their abundance is infection-related. All these suggest a possible role for these taxa in the maintenance or inducement of bladder pathologies in schistosomiasis, a disease in which inflammation is a known driver of complications. Such a role will complement other possible inducers of inflammation such as egg oviposition by the parasite. In summary, we have identified few microbial taxa that could regulate the maintenance or initiation of bladder pathologies in urogenital schistosomiasis.

A translational application of microbes is in the treatment of bladder tumours. BCG vaccine (attenuated *Mycobacterium tuberculosis*) when injected into the bladder reduced recurrence and progression in half of bladder cancer patients under study [57]. Administration of *L*. *casei* or *L*. *rhamnosus* in rodent models decreased progression of bladder tumour cells [58]. Both studies [57,58] were concerned with bladder tumours that were not due to schistosomiasis. We hypothesize that in *S*. *haematobium* induced bladder cancer, strategies to deplete these bacteria taxa combined with BCG vaccine might improve efficacy of treatment at least in some individuals.

Another taxon consistently associated with infection state in our data is the genus *Lactobacillus* (up to family and order level). High *Lactobacillus* proportion has been identified in both urine and vaginal microbiome studies, though it tends to be more prevalent in females [41]. This was far from the case in the present study. Rather, our data suggests that the microbiome in our study population belong to the ‘diverse’ or low *Lactobacillus* group urinary microbiome. As in the current study in which *Lactobacillus* is associated with infected samples, another study [19] also reported increased abundance of this genus in interstitial cystitis. Our study supports the emergent theory that rather than view the whole genus as symbiotic, certain species are more frequent in disease state, for instance, *L*. *gasseri* [39], and could probably be considered pathobiont. A proposed mechanism to explain these observations is that *Lactobacillus* tolerance to common anti-bacterial compounds allows it to multiply easily and invade tissues to cause inflammatory changes [59].

Some of the significant genera found in the present study have been previously discovered to be associated with healthy or disease state in urine microbiome studies. *Dialister* and *Gemella* were previously associated with STI [41]. *Dialister* and *Facklamia* were more frequent in incontinence, and Enterobacteriaceae and *Staphylococcus* were more frequent in healthy controls [39]. *Enterococcus* associated with cystitis [43], but *Dialister* and *Staphylococcus* have been found in healthy female urine [19]. Our data agree with these earlier studies in the sense that urine microbial genera found to be frequent in a disease state by earlier authors—*Facklamia* and *Enterococcus* were also associated with urogenital schistosomiasis. The aforementioned studies indicated that *Dialister* and *Gemella* may occur frequently in either disease or healthy state, but in the current study they were more frequent in infection. Other genera previously described in urine samples such as *Corynebacterium* and *Atopobium* were all observed in our data, but did not associate with disease or healthy state. The current study has helped to confirm earlier studies on the frequency of some urinary microbial genera in disease state.

Some of the significant taxa in the microbial community abundant in control samples (whether non-infected, non-pathology or both) are a mixture of pathobionts as well as beneficial and protective species. Enterobacteriaceae have abilities in tolerance of inflammation and redox stress; *Collimonas* (anti-fungal); *Weissella* (probiotic properties, anti-fungal, antibiotic, stress resistance); *Janthinobacterium* (antibiotic, anti-fungal, anti-tumour, stress tolerance); *Trabulsiella* (type IV secretory system to prevent competition) and unclassified Lactobacillales (lactic acid bacteria group)[60–66]. Thus, even in the absence of high proportions of *Lactobacillus sp*, there could be beneficial microbes in the urinary tract. Whether this is unique to our study population or not requires further investigation. In summary, it may be said that bacterial genera with high tolerance, antibiotic and anti-fungal properties (including lactic acid bacteria), were significant residents in control samples. As the results show, some other beneficial species may occur even in advanced cases and such may help to reduce the extent of damage caused.

Microbial-related KEGG Ontology (KO) pathways obtained from the analysis of imputed metagenomes are of biological importance. The biochemical pathways are essentially predicted from our sequence data and the proteins involved may not be translated, yet the links in predictions merit an examination. It appears that pathways to enhance proliferation and rapid multiplication of cells are increased in the infected samples compared to non-infected, and in advanced cases compared to other groups (S3A Fig, Fig 8). Hence, repair, transcriptional or translational factors and nucleotide metabolism are significantly higher in infected cases, and even higher in advanced cases (S3A Fig). Multiplication of microbes is sure to induce action from the immune system in the human host and this may aggravate infection. Hence, it is of concern that this proliferation ability increases with disease progression (i.e. from controls to infected to advanced), as our results suggest.

In addition, our results suggest decreased biosynthesis of some lipids in infected compared to controls, and in advanced cases compared to other groups. Hence, pathways significantly more abundant in non–infected cases are those that involve synthesis of several lipids involving quinones, terpenoids and steroids. An explanation for the decreased representation of lipid metabolism, synthesis, or modification pathways in infected and advanced cases could be the abundance of such molecules in the environment (since it has been reported that *S*.*haematobium* produces steroid-like lipids into the urinary tract). The question arises especially because it is known that microbes may switch on/off regulatory mechanisms in the presence of needed materials. Further research is clearly needed to explore this relationship.

Some of the significant biochemical pathways from this study have also been highlighted in earlier studies. In a review of empirical studies, Borningen et al. [67] highlighted the involvement of riboflavin metabolism in IBD (Inflammatory Bowel Disease), nucleotide and lipid metabolism in type1 diabetes. In the current study, our analysis suggests reduced riboflavin and lipid metabolism, and like IBD, urogenital schistosomiasis is also inflammation driven, leading to induced pathologies. Hence, while IBD is not a parasitic disease, it appears part of the inflammatory process involving microbes may be shared with urogenital schistosomiasis. Furthermore, there is a noticeable link among the microbial pathways whose gene abundances are reduced in infected samples. Alpha-linolenic acid is known to be produced by lactic acid bacteria, it can inhibit shikimate kinase, a precursor to the formation of chorismate (KEGG C00251) in the shikimate pathway. 4-Hydroxybenzenoate (KEGG C00156) from chorismate is used by bacteria in the biosynthesis of ubiquinone and other-terpenoid-quinone, a process more abundant in non-infected cases. Also, isochorismate (KEGG C00885) from chorismate is essential for biosynthesis of siderophore group non-ribosomal peptides, another process more abundant in non-infected cases. A product of 4-Hydroxybenzenoate, hydroquinone, in its modified form, is a byproduct in riboflavin synthesis. This linkage is probably important and could depend heavily on the shikimate pathway and chorismate.

It is known that several downstream products of chorismate are useful as antimicrobials [68], and that the ubiquinones, terpenoid-quinones and riboflavin play important roles as coenzymes in oxidative phosphorylation and electron-carrier system. Also, the siderophore group non-ribosomal peptides include several catechol-based molecules for import of iron chelation, importation of xenobiotics into the cell among other functions [69, 70]. This is in addition to the fact that some transporters are also significantly reduced. Thus, the abundance of microbial biological processes is altered in the course of urogenital schistosomiasis and bladder pathologies. An explanation for the altered abundance is that dysbiosis brought about by loss or reduction of protective, beneficial bacteria creates a more stressful and less controlled microbiome during urogenital schistosomiasis infection.

The use of midstream urine as a representative of the bladder surface from which it is discharged, is not without disagreement in literature [43, 71]. The arguments about the methods are fuelled by the possibility of contamination especially in samples from females or the elderly; hence contaminant removal is crucial. In this study, none of the elderly participants had any impairment and with proper instructions to participants, we believe that contamination was minimal. Hence, the samples analyzed in this study comprise the bladder surface microbiome. Also, in this study, virtually all genera reported from studies using transurethral catheterization to sample bladder microbiome [39] were observed. In addition, given that other forms of bladder pathology exist, a vast majority of the pathology cases recorded here are induced by urogenital schistosomiasis infection. In our study locality, bladder pathology was extensively associated with urogenital schistosomiasis [9]. It is also possible the pathology cases with no infection (determined by urine microscopy only), could be due to a chronic urogenital schistosomiasis infection in which egg shedding reduces progressively with occurrence of pathologies.

To summarize, while the current study substantially reports differences in the urinary tract microbiome between persons infected with urogenital schistosomiasis and healthy persons, and between persons having bladder pathologies due to urogenital schistosomiasis and persons with urogenital schistosomiasis infection alone, there are a few possible limitations. It remains to be confirmed if there is long-term stability in the microbiome observed, although the infected cases in the present study can be classified as chronic infection. Another limitation may be the small sample size.

## Conclusion

In this study, we examined the urinary microbiome in urinary or urogenital schistosomiasis and its induced bladder pathologies. We found that the urinary tract microbiome of the whole study population itself differs from earlier studies elsewhere. We also detected several beneficial and stress tolerant taxa in control cases, and immune-stimulatory taxa in urogenital schistosomiasis infected and urogenital schistosomiasis associated bladder pathology cases. In the microenvironment of the bladder and urinary tract, these changes probably cause lower chorismate production, synthesis of some lipids and promote self-proliferation. The urinary microbiome is a factor to be considered in developing biomarkers, diagnostic tools, and new treatment for urogenital schistosomiasis and induced bladder pathologies.

## Supporting information

S1 Fig Proportions of selected urine microbiome taxa in different states of urogenital schistosomiasis and controls. Differences in proportions were significant (p<0.05) (A) Clostridiales (B) Pseudomonadaceae, (C) Enterobacteriaceae, (D) Lactobacillus.
(TIF)

S2 Fig (A) Changes in the urine microbiome of urogenital schistosomiasis (infected) compared to controls (non-infected) with rarefaction of samples prior to analysis. (B) Differential abundance in the urine microbiome of persons with urogenital schistosomiasis infection without bladder pathology (infection-only) and pathology without schistosomiasis infection (pathology-only) (FDR<0.05).Differential abundance was measured with LogFC, the log2 of the number of times the sequences belonging to a genus (or family) are more numerous in one group relative to the other. Circle on a vertical line represents a bacterial genus or family colored by their phylum and the genus or family is named at the end of the line. More than one circle on a vertical line represent species of the same genus. The genus is labeled on the x-axis. UC represents a genus whose identity could not be completely confirmed, but with known family or order. logFC is the logarithm of the fold change between two groups. Abundant microbes in each of the two groups are presented on either side of the middle zero line.
(TIF)

S3 Fig Predicted biochemical changes due to microbiome differences in (A) urogenital schistosomiasis induced bladder pathology (advanced) and urogenital schistosomiasis infection alone (infection), (B) urogenital schistosomiasis induced bladder pathology (advanced) and pathology without infection (b.path), (C) urogenital schistosomiasis induced bladder pathology (advanced) and healthy controls (control).
(TIF)
