# Correction: The microbiome in urogenital schistosomiasis and induced bladder pathologies

**DOI:** 10.1371/journal.pntd.0006067

**Published:** 2017-11-15

**Authors:** Adewale S. Adebayo, Mangesh Survayanshi, Shrikanth Bhute, Atinuke M. Agunloye, Raphael D. Isokpehi, Chiaka I. Anumudu, Yogesh S. Shouche

The second and third authors’ names are spelled incorrectly. The correct names are: Mangesh Vasant Suryavanshi and Shrikant Bhute. The correct citation is: Adebayo AS, Suryavanshi MV, Bhute S, Agunloye AM, Isokpehi RD, Anumudu CI, et al. (2017) The microbiome in urogenital schistosomiasis and induced bladder pathologies. PLoS Negl Trop Dis11(8): e0005826. https://doi.org/10.1371/journal.pntd.0005826
