# Supplementary figures and images for: The microbiome in urogenital schistosomiasis and induced bladder pathologies
Source: PLoS Negl Trop Dis. 2017 Aug 9;11(8):e0005826. doi: 10.1371/journal.pntd.0005826 (PMC5565189; doi:10.1371/journal.pntd.0005826)

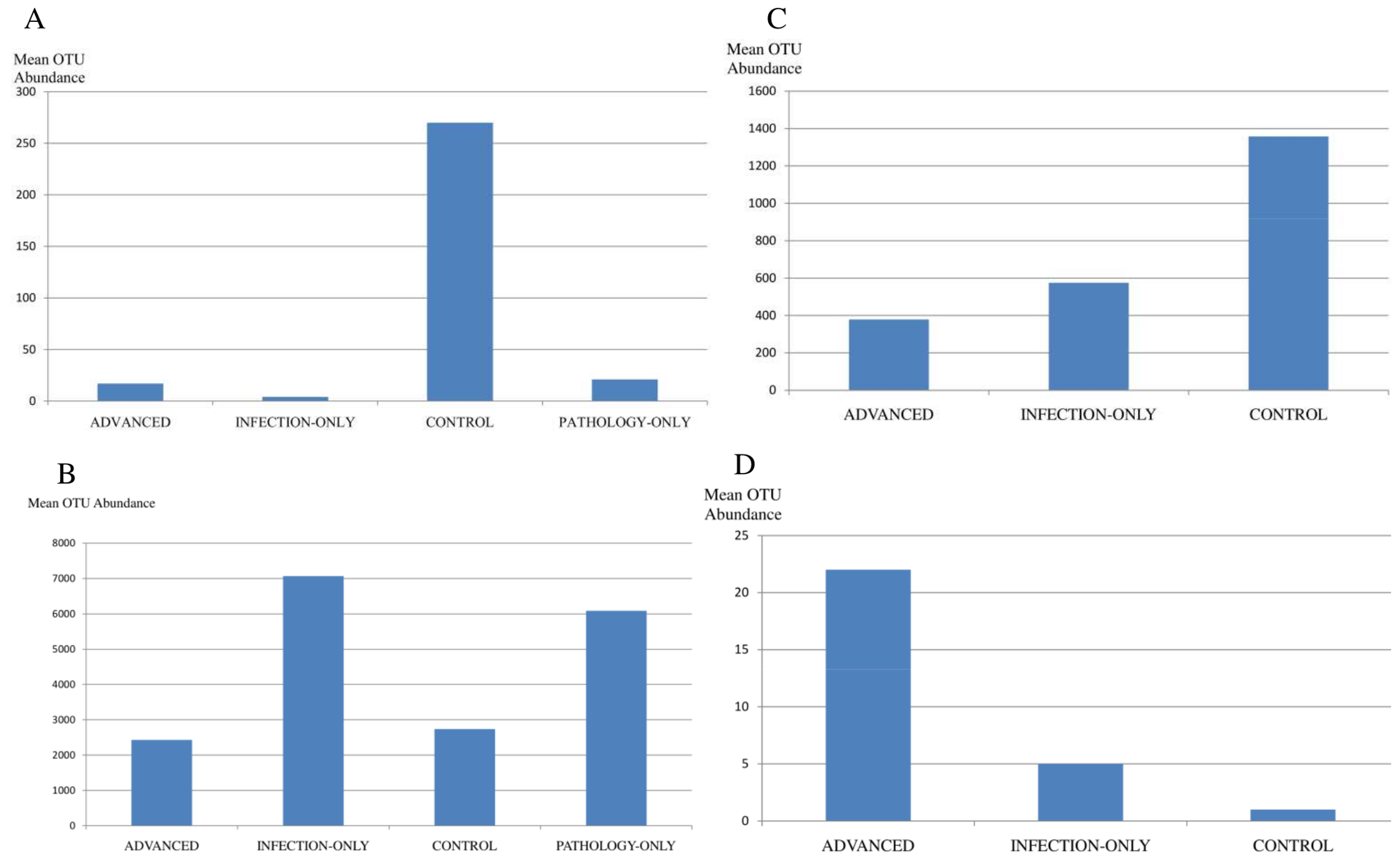

Supplement: S1 Fig — Differences in proportions were significant (p<0.05) (A) Clostridiales (B) Pseudomonadaceae, (C) Enterobacteriaceae, (D) Lactobacillus. (TIF) [file pntd.0005826.s001.tif]

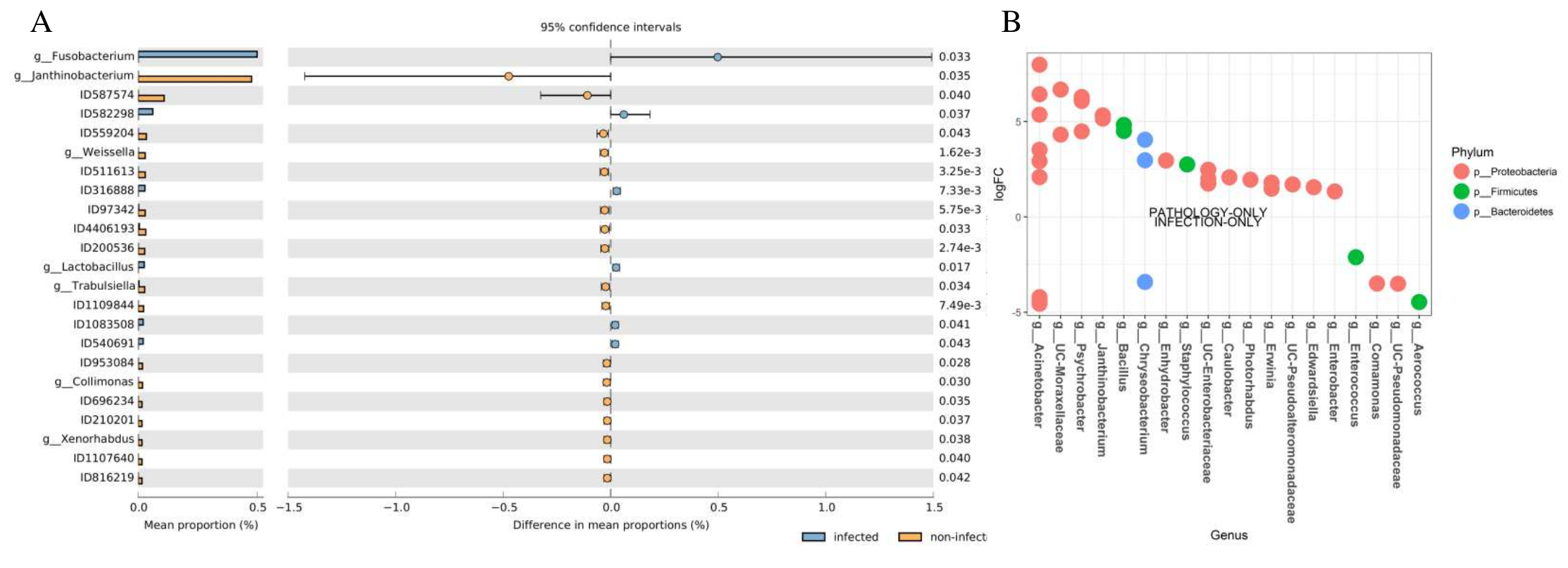

Supplement: S2 Fig — (A) Changes in the urine microbiome of urogenital schistosomiasis (infected) compared to controls (non-infected) with rarefaction of samples prior to analysis. (B) Differential abundance in the urine microbiome of persons with urogenital schistosomiasis infection without bladder pathology (infection-only) and pathology without schistosomiasis infection (pathology-only) (FDR<0.05).Differential abundance was measured with LogFC, the log2 of the number of times the sequences belonging to a genus (or family) are more numerous in one group relative to the other. Circle on a vertical line represents a bacterial genus or family colored by their phylum and the genus or family is named at the end of the line. More than one circle on a vertical line represent species of the same genus. The genus is labeled on the x-axis. UC represents a genus whose identity could not be completely confirmed, but with known family or order. logFC is the logarithm of the fold change between two groups. Abundant microbes in each of the two groups are presented on either side of the middle zero line. (TIF) [file pntd.0005826.s002.tif]

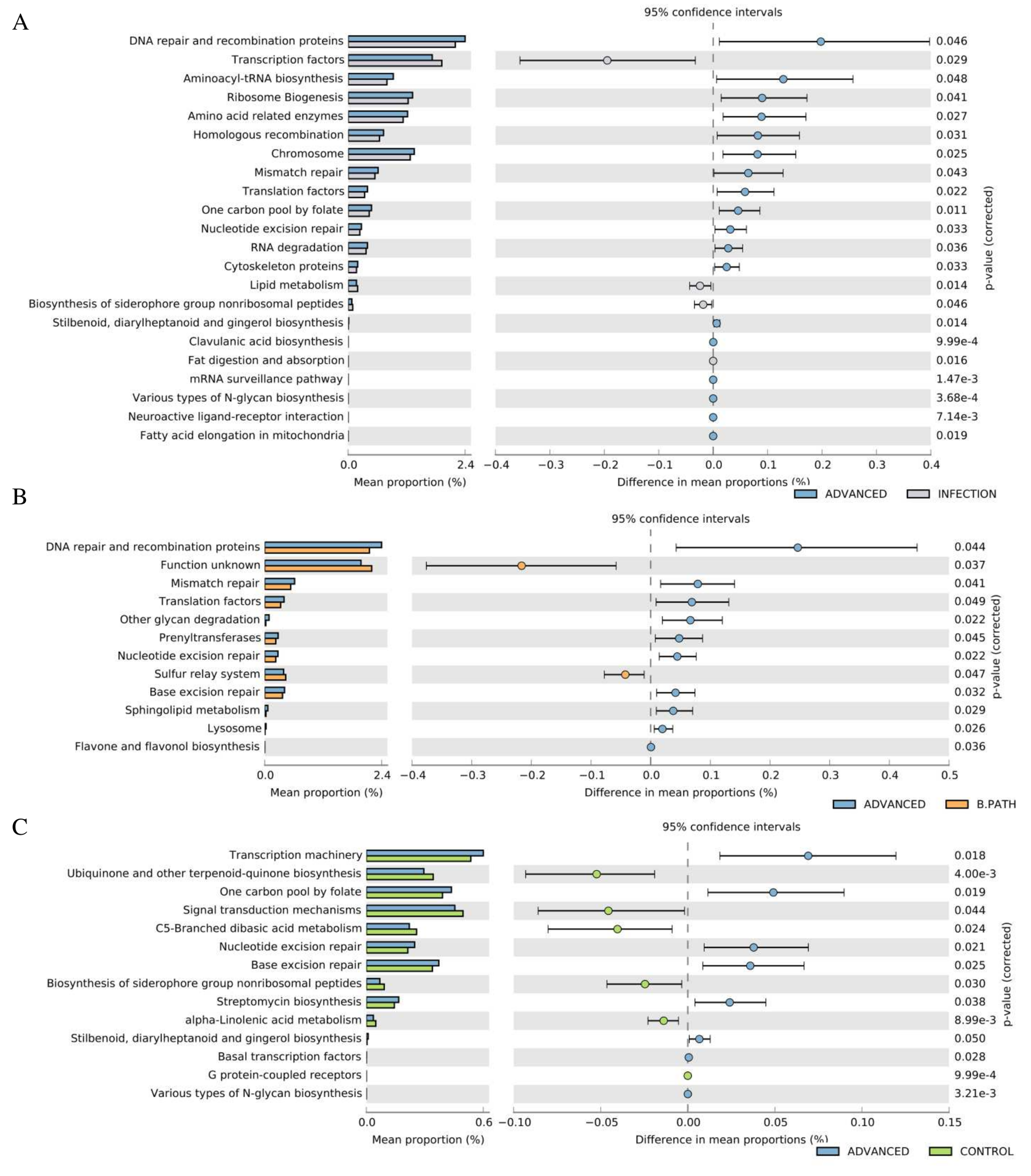

Supplement: S3 Fig — Predicted biochemical changes due to microbiome differences in (A) urogenital schistosomiasis induced bladder pathology (advanced) and urogenital schistosomiasis infection alone (infection), (B) urogenital schistosomiasis induced bladder pathology (advanced) and pathology without infection (b.path), (C) urogenital schistosomiasis induced bladder pathology (advanced) and healthy controls (control). (TIF) [file pntd.0005826.s003.tif]
